# A narrative literature review about alpha‐lipoic acid role in dry eye and ocular surface disease

**DOI:** 10.1111/aos.17486

**Published:** 2025-04-10

**Authors:** Antonio J. Mateo Orobia, José Manuel Benítez del Castillo, Margarita Calonge, Christophe Baudouin, Marc Labetoulle

**Affiliations:** ^1^ Hospital Universitario Miguel Servet Zaragoza Instituto Oftalmológico Biotech‐Visión. Quirónsalud Zaragoza Zaragoza Spain; ^2^ Universidad Complutense, Hospital Clínico San Carlos Madrid, Ocumed Clínica Oftalmológica, Clínica Rementería Madrid Spain; ^3^ Universidad de Valladolid, Instituto Universitario de Oftalmología Aplicada Valladolid (IOBA) Valladolid Spain; ^4^ Department of Ophthalmology Quinze‐Vingts National OphthalmologyHospital and Vision Institute Paris France; ^5^ Service d'Ophtalmologie, Hôpital Bicêtre, Assistance Publique‐Hôpitaux de Paris Paris‐Saclay University Kremlin‐Bicêtre France

**Keywords:** alpha‐lipoic acid, antioxidants, diabetic retinopathy, dry eye disease, ocular surface disease, prevention

## Abstract

Ocular surface diseases (OSD) include various conditions that affect the eye's surface, causing discomfort and pain. One such condition, dry eye disease (DED), is a multifactorial disorder that significantly impacts patients' quality of life, with prevalence rates ranging from 5% to 50% and higher incidence in women. DED involves tear film instability, inflammation and neurosensory abnormalities, making its management challenging due to diverse underlying mechanisms. Conventional treatments typically focus on symptom relief, but new approaches targeting the disease's pathogenesis are emerging. Alpha‐lipoic acid (ALA) is gaining attention for its potential in treating OSD and DED. ALA acts as a potent antioxidant, neutralizing reactive oxygen species. It protects cell membranes by interacting with vitamin C and glutathione, potentially recycling vitamin E. Its antioxidative properties are particularly relevant in meibomian gland dysfunction, a condition implicated in DED. By scavenging free radicals and modulating redox status in the meibomian glands, ALA can reduce oxidative damage, preserve glandular function and decrease inflammation. In diabetic patients with DED, ALA administration has been found to improve tear film parameters, reduce corneal defects, enhance antioxidant status and potentially prevent diabetic retinopathy and keratopathy. Its therapeutic effects on neurosensory abnormalities, especially in diabetic polyneuropathy and other neuropathies, are primarily due to its antioxidant, anti‐inflammatory and metal‐chelating properties. In summary, ALA holds promise as a therapeutic agent for DED and OSD and could be a promising treatment option for diabetic retinopathy and keratopathy, although further research is needed to confirm its efficacy.

## 1 INTRODUCTION

Ocular surface diseases (OSD) encompass a wide range of conditions that contribute to both ocular surface alterations and discomfort or pain. They can be triggered by systemic disorders and environmental factors that induce persistent inflammation in the ocular adnexal connective tissues, such as the conjunctiva, lacrimal gland and meibomian glands.

OSD can cause but is not limited to dry eye disease (DED); they all may, however, significantly impact the quality of life of affected patients worldwide. Prevalence rates range from 5% to 50% but can be as high as 75% among adults over age 40, with women being more often affected, likely as a consequence of decreased androgen levels and exogenous oestrogen use (Stapleton et al., 2017); (Rouen & White, 2018). DED is characterized by a complex multifactorial aetiology involving tear film instability, ocular surface inflammation and neurosensory abnormalities, leading to symptoms of ocular discomfort, visual disturbances and potential damage to the ocular surface (Craig et al., 2017).

The management of DED and OSD presents a significant clinical challenge due to the diverse underlying mechanisms contributing to these conditions. Advances in our understanding of the risk factors, aetiology and pathophysiology of DED have contributed to an evolution in treatment strategies over time (Jones et al., 2017). During the last two decades, there has been a growing realization of the important contribution of meibomian gland dysfunction (MGD) to both symptoms and signs of DED (Nelson et al., 2011). Conventional treatment options primarily focus on symptom relief through the use of artificial tears and lifestyle modifications, and also immune‐modulatory agents in the most severe cases (Thode & Latkany, 2015); (O'Neil et al., 2019). However, there is a growing interest in exploring novel and in‐between therapeutic approaches that target the underlying pathogenesis of DED and OSD, aiming for more effective and sustainable outcomes (Coco et al., 2023).

Oxidative stress has been implicated in ageing and a variety of age‐related chronic diseases, including several ocular pathologies, such as DED, cataracts, glaucoma, age‐related macular degeneration and diabetic retinopathy (Rodella et al., 2023). DED pathophysiology includes an excess of inflammatory cytokines that increase reactive oxygen species (ROS) production, leading to a disruption of the balance between the antioxidant and the prooxidant systems, damaging the healthy tear film (Dogru et al., 2018).

Many biological effectors with antioxidant potential are present in different parts of the eye. The human body endogenously produces some of them, such as glutathione, N‐acetylcysteine, alpha‐lipoic acid (ALA), coenzyme Q10 and enzymatic antioxidants. Others, such as plant‐derived polyphenols and carotenoids, vitamins B2, C, and E, zinc and selenium and omega‐3 polyunsaturated fatty acids, are obtained through the diet and are considered essential nutrients (Rodella et al., 2023). Nevertheless, when equilibrium between the production of ROS and the scavenging molecules is disrupted, treating patients with antioxidants may logically be valuable. Therapeutic strategies to decrease oxidative stress include the administration of antioxidants to scavenge ROS and the modulation of cellular signalling pathways that regulate ROS production and antioxidant defence mechanisms (Böhm et al., 2023). Several studies have shown that supplementation with oral antioxidants can improve eye clinical signs. A summary of non‐enzymatic antioxidants with evidence in eye diseases is shown in Table 1.

**TABLE 1:** Overview of non‐enzymatic antioxidants that have demonstrated efficacy in eye diseases, including their mechanisms of action and clinical relevance.

Group	Antioxidant	Eye health evidence
Lipophilic antioxidants in cellular membranes	Omega‐3 Fatty Acids, including eicosapentaenoic (EPA) and docosahexaenoic acid (DHA)	Omega‐3 Fatty Acids are enhancer factors in antioxidant defence against ROS (Heshmati et al., 2019). In the context of the ideal dietary omega‐3/omega‐6 ratio of 1:4 (Yehuda and Carasso, 1993), omega‐3 play a crucial role in modulating inflammation by inhibiting the production of pro‐inflammatory mediators like prostaglandins and leukotrienes (Calder, 2020); (Parolini, 2024). Additionally, they interfere with arachidonic acid metabolism, leading to the synthesis of less inflammatory analogues and specialized pro‐resolving mediators. This ultimately reduces the production of inflammatory cytokines and other molecules (Hernandez‐Molina et al., 2019). Several studies have shown that omega‐3 supplementation can improve symptoms of DED by reducing inflammation and improving tear quality. (Bhargava et al., 2013); (Bhargava et al., 2023); (He and Bazan, 2010); (Meng, Shi and Hong‐Yan, 2022). A Cochrane review in DED suggests a possible role for long‐chain omega‐3 supplementation in managing DED, although the evidence is uncertain and inconsistent.(Downie et al., 2019)
	Vitamin E	Vitamin E is a potent chain‐breaking antioxidant that inhibits the production of reactive oxygen species molecules when fat undergoes oxidation and during the propagation of free radical reactions (Rizvi et al., 2014). Experimental and clinical researches indicate that vitamin E supplementation can impact humoral and cell‐mediated immunity, including antibody production, lymphocyte proliferation and phagocytosis (Meydani, Han and Wu, 2005); (Mocchegiani and Malavolta, 2016). Vitamin E can reduce inflammation on the ocular surface. Some studies suggest that supplementation with vitamin E may improves ocular health (Age‐Related Eye Disease Study Research Group, 2001); (Serhan et al., 2022); (McCusker et al., 2016)
	Coenzyme Q10	Coenzyme Q10 is one of the most significant lipid antioxidants, which prevents the generation of free radicals and subsequent modifications of proteins, lipids and DNA. Studies have shown that Coenzyme Q10 can reduce oxidative stress and apoptosis in various diseases, including cancer (Dadali et al., 2021) and plays a crucial role in mitochondrial ATP synthesis by functioning as a cofactor in the mitochondrial respiratory chain, facilitating electron transfer (Münch et al., 2023). In many disease conditions connected with increased generation and the action ROS, the concentration of coenzyme Q10 in the human body decreases (Saini, 2011). There are evidences for its involvement with corneal damage and glaucoma (Ahmad et al., 2017). A combination of coenzyme Q10 with other nutrients may improve dry eye symptoms (Feher et al., 2006); (Serrano‐Morales et al., 2022). CoQ10 treatment plus hyaluronic acid show greater effectiveness in DED compared to HA alone, probably due to a prolonged of the antioxidant activity of CoQ10 on the ocular surface (Postorino et al., 2018)
	Alpha‐lipoic acid (ALA)	A detailed overview of the ALA is provided in the subsequent sections of this review
Water‐Soluble Promoters of Endogenous Antioxidant System	Vitamin C	As an antioxidant, vitamin C provides protection against oxidative stress‐induced cellular damage by scavenging of ROS that can help protect ocular cells (Traber and Stevens, 2011). Ocular symptoms of vitamin C deficiency include dry eyes, scleral icterus and subconjunctival haemorrhage (Serhan et al., 2022). While there is less specific evidence regarding its effectiveness in treating DED, adequate intake of vitamin C is known to be important for overall ocular health (McCusker et al., 2016)
	Zinc	The ability of zinc to retard oxidative processes has been recognized for many years (Powell, 2000); (Marreiro et al., 2017); (Lee, 2018). Zinc is an inhibitor of nicotinamide adenine dinucleotide phosphateoxidase, which results in decreased generation of ROS (Prasad, 2014), tear film stability and decrease of dry eye complaints.(Perényi et al., 2017); (Park, Hwang and Shin, 2022)
	Selenium	In recent years, Selenium research has attracted tremendous interest because of its important role in antioxidant selenoproteins for protection against oxidative stress initiated by excess ROS and reactive nitrogen species (Tinggi, 2008); (Björklund et al., 2022); (Zakeri et al., 2021). DED is related to oxidative stress and selenium compounds are probably candidates for treatment of dry eye by regulating oxidative stress in the corneal epithelium (Higuchi et al., 2012); (Higuchi et al., 2016); (Pasricha, 2023)
	N‐acetyl cysteine	The cysteine prodrug N‐acetyl cysteine (NAC) is widely used as a pharmacological antioxidant and cytoprotectant. N‐acetyl cysteine has shown promising anti‐inflammatory effects in various conditions as Parkinson disease (Kaya et al., 2023), obesity (Sztolsztener et al., 2023) or rheumatoid arthritis (Jamali et al., 2021). It has been reported to lower endogenous oxidant levels and to protect cells against a wide range of pro‐oxidative insults (Ezerina et al., 2018); (Zhitkovich, 2019); (Tenório et al., 2021). A systematic search of peer‐reviewed articles identified 106 references including in vitro, in vivo and clinical studies on the use of NAC in the treatment of ocular diseases (Eghtedari et al., 2022). NAC appears to be a safe and effective treatment of DED (Nepp et al., 2020); (Messina and Dua, 2019)

Riboflavin	When combined with ultraviolet (UV) light, riboflavin has demonstrated various protective and antimicrobial properties (Zhu, Li and Wang, 2020). Riboflavin can protect the body against oxidative stress, especially lipid peroxidation and reperfusion oxidative injury. The mechanisms by which riboflavin protects the body against oxidative stress may be attributed to the glutathione redox cycle and also to other possible mechanisms such as the conversion of reduced riboflavin to the oxidized form. (Ashoori and Saedisomeolia, 2014); (Olfat, Ashoori and Saedisomeolia, 2022) Riboflavin also favours the absorption and concentration of ultraviolet light in the anterior half of the corneal stroma. Severe riboflavin deficiency plays an important role in the aetiology of cataract (Mazzotta, Caragiuli and Caporossi, 2014). A vitamin complex formulation containing riboflavin is effective in stabilizing tear stability and alleviating symptoms in patients with intractable dry eye (Park, Hwang and Shin, 2022)
Carotenoids	Lutein and Zeaxanthin	Lutein and zeaxanthin are selectively concentrated in vision‐related tissues (the eye and brain) are protective against oxidative stress (Johnson, 2014); (Choo et al., 2022). Both of these carotenoids can be found in the macula and retina of your eyes, where they help filter potentially harmful blue light, protecting your eyes from damage (Kijlstra et al., 2012). Several studies suggest that these plant compounds may prevent cataracts and prevent or slow down the progression of AMD (Liu et al., 2014); (Jia et al., 2017)
	Astaxanthin	Astaxanthin stretches through the bilayer membrane, providing protection against oxidative stress by scavenging reactive oxygen species in both the inner and outer layers of the cellular membrane. Preclinical and clinical evidences support the potential use of astaxanthin in the prevention and treatment of a number of ocular diseases (Giannaccare et al., 2020); (Yang and Wang, 2022)
	Beta‐carotene	As an antioxidant, betacarotene inhibits free radical damage to DNA and is typically associated with ocular health (Age‐Related Eye Disease Study Research Group, 2001). High concentration of beta‐carotene may reduce the risk cataract development (Choo et al., 2022). The use of beta‐carotene has been replaced with lutein and zeaxanthin (Lawrenson and Grzybowski, 2015). Oral administration of a vitamin complex formulation, including beta‐carotene was effective in stabilizing tear stability and alleviating symptoms in patients with intractable dry eye (Park, Hwang and Shin, 2022)
Polyphenols	Polyphenols	Polyphenols are strong antioxidants that complement and add to the functions of antioxidant vitamins and enzymes as a defence against oxidative stress caused by excess reactive oxygen species (Tsao, 2010). Polyphenols are also thought to reduce inflammation, which is thought to be the root cause of many chronic illnesses (Hunter, 2012). Polyphenols play a significant role in vision physiology and antioxidant protection of the eye, with potential effects in degenerative retinal diseases and in age‐related eye diseases and dry eye disease (Srinivasan, 2014); (Fernandez‐Gonzalez, Mas‐Sanchez and Garriga, 2021); (Bungau et al., 2019); (Favero et al., 2021)
	Flavonoids	Flavonoids are phenolic substances isolated from a wide range of vascular plants, act as antioxidants and antimicrobials (Pietta, 2000). Flavonoids are associated with a broad spectrum of health‐promoting effects (Panche, Diwan and Chandra, 2016). Flavonoids show promise as potential complementary treatment for allergic diseases because of their anti‐inflammatory and antiallergic properties. Several studies implementing ocular and systemic application of these compounds show potential in becoming adjuvant treatment strategies for improving quality of life while also managing ocular surface disease processes (Bielory and Tabliago, 2020); (Davinelli et al., 2021)
	Catechins	Catechins appear to be able to both generate and to scavenge free radicals and show their beneficial effects due to a combination of both mechanisms (Bernatoniene and Kopustinskiene, 2018); (Sheng, Sun and Tang, 2023). Studies have shown therapeutical benefits in various ocular diseases (Feng and Zhang, 2023); (Li et al., 2023); (Favero et al., 2021); (Ng et al., 2023); (Lee and Dana, 2010)
	Anthocyanins	Anthocyanins have powerful antioxidant properties, and the content of anthocyanin pigment directly correlates with antioxidant activity of plants (Bagchi et al., 2004); (Kalkan Yildirim, 2006); (Zafra‐Stone et al., 2007); (Chu et al., 2011). Blueberry can improve tear secretion and plasmatic antioxidant potential in subjects suffering from DED symptoms (Riva et al., 2017); (Yu et al., 2023); (Wakana et al., 2007). Some studies have provided evidences that oligomers of anthocyanins are safe and effective in dry eye disease, improving the tear break‐up time, intraocular pressure, ocular surface disease and patient symptomatology (Fan et al., 2023); (Wattanathorn et al., 2023); (Chiang et al., 2023)

	Curcumin	Curcumin, through its chemical structure is attributed to many properties, such as antioxidant and anti‐inflammatory among others (Menon and Sudheer, 2007); (Alizadeh and Kheirouri, 2019); (Jakubczyk et al., 2020). Curcumin supplements alone can reduce the signs and symptoms of DED as well as the frequency of tear substitute usage (Jurenka, 2009); (Aggarwal and Sung, 2009); (Borselli et al., 2023); (Chung et al., 2012); (Radomska‐Leśniewska et al., 2019)
	Resveratrol	Resveratrol may act as a nutritional supplement with a wide range of pharmacological effects, including cellular defensive action against oxidative stress (Marques, Marcus and Morris, 2009); (Salehi et al., 2018). Studies have also explored the beneficial effects of resveratrol in DED, making it as a promising therapeutic molecule (Shetty et al., 2023); (Bryl et al., 2022)
Manuka honey	Manuka honey is characterized by a high content of polyphenols and relatively high antioxidant capacity (Gośliński, Nowak and Kłębukowska, 2020); (Hunter et al., 2021); (Bazaid et al., 2022). Manuka honey has demonstrated promising results for the treatment of DED; however, further high‐quality randomized clinical trials are required to confirm the efficacy and safety of the use of Manuka honey in the treatment of DED.(Hu et al., 2023)
Other antioxidants	Crocus Sativus (Saffron)	Saffron and its main constituents (crocin, picrocrocin and safranal) (Khazdair et al., 2015) have antioxidant, anti‐inflammation and antiapoptotic characteristics presenting significant protective effects in age‐related diseases (Samarghandian, Farkhondeh and Zeinali, 2020); (Rahiman et al., 2018); (Abou‐Hany et al., 2018). There are several clinical studies that have assessed the impact of oral supplementation with saffron or one of its constituents on vision‐related parameters in adults with ocular diseases (Falsini et al., 2010); (Piccardi et al., 2012); (Lashay et al., 2016); (Broadhead et al., 2019); (Heitmar, Brown and Kyrou, 2019) and specifically in DED (Yousefi‐Manesh et al., 2022)
	Castor Oil	Ricinus communis (commonly known as castor oil plant) belongs to family Euphorbiaceae. Free radical scavenging and anti‐inflammatory activities of methanolic extract of Ricinus communis roots have been studied (Ilavarasan, Mallika and Venkataraman, 2006); (Iqbal et al., 2012). Studies reveal that castor oil applied topically to the ocular surface has a prolonged residence time, facilitating increased tear film lipid layer thickness, stability, improved ocular surface staining and symptoms (Sandford, Muntz and Craig, 2021)

One such emerging therapeutic agent is ALA, a naturally occurring compound with potent antioxidant and anti‐inflammatory properties (Zhang et al., 2023); (Capece et al., 2022); (Ambrosi et al., 2018); (Packer et al., 1995). ALA has gained attention in various medical fields for its potential therapeutic effects in conditions characterized by oxidative stress and inflammation, including diabetes, neurodegenerative disorders and cardiovascular diseases (Viana et al., 2022); (Ziegler & Gries, 1997); (Bossio et al., 2023); (Capece et al., 2022); (Hsieh et al., 2023). Given the evidence implicating oxidative stress and inflammation in the pathogenesis of DED and OSD, ALA has garnered interest as a potential adjunctive therapy for these ocular conditions.

The purpose of this narrative review is to evaluate the existing evidence regarding the role of ALA in the management of DED and OSD. By systematically analysing preclinical studies, clinical trials and observational research, we aimed to elucidate the mechanisms underlying ALA's therapeutic effects on ocular surface health and assess its potential clinical efficacy and safety profile in the context of DED and OSD. Furthermore, through this comprehensive review, we aimed to provide valuable insights into the potential role of ALA as a novel therapeutic option for DED and OSD, thereby contributing to the development of more effective strategies for the management of these prevalent ocular conditions.

## 2 METHODOLOGY

In this literature review, we conducted a narrative analysis, covering a wide range of topics by incorporating studies of varying complexity and design (Grant & Booth, 2009). The search was conducted between December 2023 and May 2024 across PubMed, Scopus, Web of Science and Google Scholar databases. Our strategy involved combining keywords related to ALA and ocular diseases, additionally, we identified subject headings variations in thesaurus and indexing terms. Example search terms included: ‘Alpha Lipoic Acid’, ‘ALA’, ‘Thioctic Acid’, ocular surface disease’, ‘dry eye disease’, ‘ocular health’, ‘cataract’, ‘glaucoma’, ‘diabetic retinopathy’, ‘age‐related macular degeneration’, ‘oxidative stress’, ‘neuroprotection’, ‘neurosensorial’, ‘antioxidant’, ‘anti‐inflammatory’ and ‘therapeutic uses’. These terms were used individually or in combination with Boolean operators (AND, OR) to capture all relevant studies.

### 2.1 Study selection process

The selection process followed a structured, step‐by‐step approach to ensure that only relevant, high‐quality studies were included.

#### 2.1.1 Initial database search and screening

Databases were searched using relevant keywords, including studies on ALA's role in diseases like diabetes, neurodegeneration and systemic inflammation, to explore cross‐cutting mechanisms. Book chapters on ALA were also included. Duplicate articles were removed to refine the pool.

#### 2.1.2 Title and abstract screening

Titles were reviewed to exclude studies unrelated to ALA or antioxidants in ocular diseases. Relevant studies from other fields were retained if they provided insights into ALA's antioxidant, anti‐inflammatory or neuroprotective effects. Abstracts were screened for relevance to oxidative stress and inflammation themes.

#### 2.1.3 Application of inclusion and exclusion criteria

Inclusion Criteria:

- Articles with levels of evidence I–III used in NICE guidance (Developing NICE guidelines: the manual, 2014).
- Peer‐reviewed trials, preclinical studies and reviews on ALA in ocular diseases or related mechanisms.
- Studies on other antioxidants in eye disorders.
- Articles published in the last 15 years, with key older studies included.

Exclusion Criteria:

- Articles not focused on ALA or its mechanisms.
- Articles with level IV of evidence according to NICE guidance (Eccles and Mason, 2001) and poor methodological quality.

#### 2.1.4 Full‐text review

Full‐text review of 300 articles assessed their relevance to ALA in ocular diseases and mechanisms such as neuroprotection and inflammation reduction. Non‐ocular studies offering transferable insights (e.g., ALA's role in diabetic retinopathy or neurodegeneration) were included. Studies with significant flaws or limited relevance were excluded.

#### 2.1.5 Prioritizing high‐impact studies and comprehensive themes

Priority was given to studies on ALA in ocular surface disease. High‐impact articles on ALA's mechanisms in other conditions were included if relevant. Expert review ensured comprehensive coverage.

#### 2.1.6 Backward reference checking

References of key papers were reviewed to identify additional relevant studies.

#### 2.1.7 Final selection

201 high‐quality articles, including ocular‐specific and broader research, were selected to provide a comprehensive view of ALA's role in reducing oxidative stress, inflammation and neurodegeneration.

**FIGURE 1:**
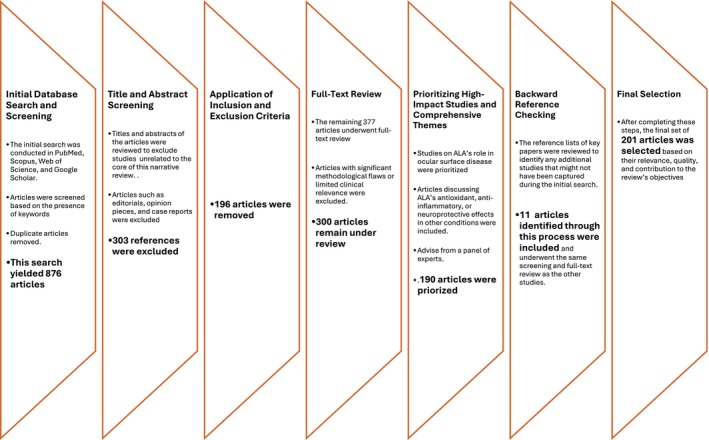
Study selection process to ensure the inclusion of high‐quality, relevant studies on ALA's role in ocular diseases.

The process is summarized in Figure 1.

### 2.2 Synthesis of evidence

The synthesis of evidence was organized around key themes, providing a narrative summary of the data collected. Key data points from each study were extracted to ensure a comprehensive understanding of ALA's role in ocular diseases. These data were categorized into the following areas:

- Antioxidant Properties: Studies examine how ALA reduces oxidative stress in ocular tissues, helping to prevent or mitigate damage to retinal cells, lens proteins and other ocular structures.
- Anti‐inflammatory Effects: Data on ALA's role in reducing inflammation in the eye, particularly in conditions like DED, OSD, uveitis, glaucoma and diabetic retinopathy.
- Neuroprotection: Evidence highlighting ALA's protective effects on retinal ganglion cells, photoreceptors and the optic nerve, especially in neurodegenerative ocular diseases. Additionally, studies supporting ALA's role in addressing other neurosensory abnormalities were included.

## 3 RESULTS

### 3.1 ALA and oxidative stress

ALA plays an essential role in mitochondrial dehydrogenase reactions, gaining considerable attention as an antioxidant. Lipoate or its reduced form, dihydrolipoate, reacts with ROS such as superoxide radicals, hydroxyl radicals, hypochlorous acid, peroxyl radicals and singlet oxygen. It also protects membranes by interacting with vitamin C and glutathione, which may in turn recycle vitamin E (Packer et al., 1995); (Ajith, 2020). In addition to its antioxidant activities, dihydrolipoate may exert prooxidant actions through the reduction of iron and copper. ALA administration has been shown to be beneficial in a number of oxidative stress models, such as ischemia–reperfusion injury (Qi et al., 2022), diabetes (both ALA and dihydrolipoic acid exhibit hydrophobic binding to proteins such as albumin, which can prevent glycation reactions); (Zhang et al., 2023); (Capece et al., 2022), cataract formation (Khan et al., 2017), human immunodeficiency virus activation (Patrick, 2002), transplantation (Ambrosi et al., 2018), neurodegeneration (Memudu & Adanike, 2022); (dos Santos et al., 2019), neuropathic pain (Viana et al., 2022) and radiation injury (Sheikholeslami et al., 2021). Furthermore, lipoate can function as a redox regulator of proteins such as myoglobin, prolactin, thioredoxin and the NF‐kappa B transcription factor (Lee & Hughes, 2002). The antioxidant effects of ALA were found in rats subjected to sepsis in which ALA was able to decrease oxidative stress in the kidney and other organs, including the liver and heart (Petronilho et al., 2016).

In addition, ALA demonstrated anti‐inflammatory properties by downregulating the expression of redox‐sensitive pro‐inflammatory proteins, including tissue necrosis factor alpha (TNF‐α), interleukin‐6 (IL‐6) and inducible nitric oxide synthase (Skibska et al., 2023).

**FIGURE 2:**
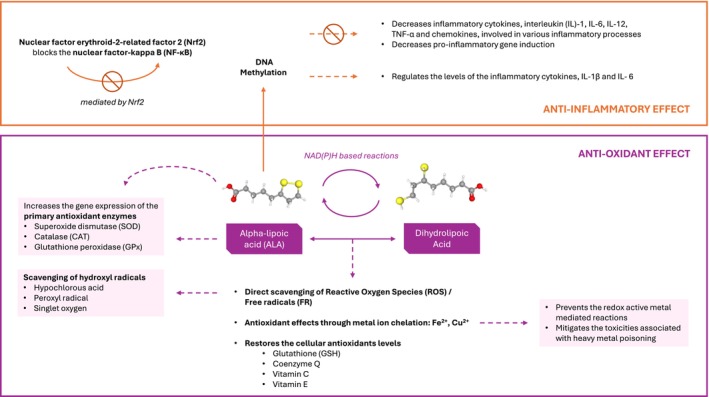
Mechanism of action of ALA including anti‐inflammatory and antioxidant effects.

A summary of the mechanism of action of ALA is shown in Figure 2.

### 3.2 ALA and neurosensory abnormalities

ALA has been extensively studied for its therapeutic effects on neurosensory abnormalities, particularly in conditions like diabetic polyneuropathy and other forms of neuropathy. Its efficacy is primarily attributed to its antioxidant, anti‐inflammatory and metal chelating properties, which play a crucial role in managing conditions associated with oxidative stress and inflammation (Fasipe et al., 2023) Research suggests that a dose of 1800 mg daily for 12 weeks can significantly improve neurosensory abnormalities in diabetic polyneuropathy (Gu et al., 2010), while a 600 mg oral dose daily for up to 5 weeks can offer benefits without significant side effects (Garcia‐Alcala et al., 2015). Additionally, a 600 mg oral dose once daily for 5 weeks has been found to improve neuropathic symptoms and deficits in patients with distal symmetric polyneuropathy; an oral dose of 600 mg once daily appears to provide the optimum risk‐to‐benefit ratio (Ziegler & Gries, 1997); (McIlduff & Rutkove, 2011). ALA's liquid formulations are associated with greater plasma concentration and bioavailability as compared to its solidified dosage form. Thus, improved formulations can increase both ALA absorption and bioavailability, leading to a rise in therapeutic efficacy (Salehi et al., 2019).

In the mechanism underlying the improvement of positive neuropathic sensory symptoms, antioxidant activity appears to be a likely mechanism. There is accumulating evidence from experimental animal and tissue culture studies that full radical‐mediated oxidative stress is implicated in the pathogenesis of diabetic polyneuropathy by inducing neurovascular defects that result in endoneurial hypoxia and subsequent nerve dysfunction (Kellogg & Pop‐Busui, 2005). Administration of physiological antioxidants, including ALA, provides a basis for a potential therapeutic effect. Diabetic peripheral nerves demonstrate footprints of oxidative stress and respond to treatment with ALA (Piddubna et al., 2020). Specifically, in diabetic peripheral neuropathy, a common neurological disorder among patients with diabetes mellitus, ALA has demonstrated significant clinical usefulness. Controlled clinical trials have shown that ALA treatment leads to an amelioration in nerve conduction velocity scores, a clinically significant reduction of neuropathic pain and improvements in serum triglycerides and insulin sensitivity (Sementina et al., 2022).

Moreover, ALA has also shown neuroprotective effects in chronic constriction injury models of neuropathic pain, indicating its potential as a treatment for neuropathic pain caused by peripheral nerve injury (Foster, 2007).

Its ability to cross the blood–brain barrier due to its lipophilic nature also makes it a promising agent in the treatment of central nervous system disorders, potentially influencing the development of diseases like multiple sclerosis and Alzheimer's (AlMomen & Blaurock‐Busch, 2022). Research into the clinical use of ALA for psychiatric and neurological conditions has highlighted its effectiveness in improving symptoms of schizophrenia, preventing Alzheimer's disease progression and reversing clinical parameters in stroke patients (de Sousa et al., 2019).

Additionally, ALA's analgesic potential in managing neuropathic pain, a prevalent form of chronic pain caused by nervous system diseases, has been demonstrated through various preclinical and clinical studies (Viana et al., 2022). Its broad mechanism of action, including the prevention of ROS formation and neuronal damage, supports its use in treating neurological and psychiatric diseases (Cekici & Bakirhan, 2018).

ALA has been shown to improve oxidative stress markers in cerebral ischemia–reperfusion models; its effects can vary depending on the administered concentration (Qi et al., 2022); (Dong et al., 2015).

Meta‐analyses have also shown ALA's efficacy in treating different types of pain, including headache, carpal tunnel syndrome and burning mouth syndrome, with specific efficacy in diabetic polyneuropathy pain symptoms (Cassanego et al., 2022). Lastly, ALA's consumption has been linked to beneficial effects on lipid metabolism, endothelial activation and its anti‐inflammatory, antithrombotic and anti‐atherosclerotic properties, particularly in patients with diabetic neuropathy (Serhiyenko, 2018).

The combination of ALA with other treatments, such as gamma‐linolenic acid (GLA), has been found to have a synergistic effect on neurovascular function, further enhancing its therapeutic potential (Cameron & Cotter, 2000). However, the optimal dose and duration of ALA treatment remain subjects of ongoing research, with studies suggesting that a dose of 600 mg once daily may offer the best risk‐to‐benefit ratio (Wang et al., 2021). Despite its benefits, ALA's efficacy can be influenced by factors such as the severity of the neuropathy and the duration of the disease (Ziegler et al., 2006).

Methylcobalamin (MC) and ALA combination has been extensively studied for its efficacy in treating diabetic peripheral neuropathy. Clinical trials have demonstrated significant improvements in neuropathy symptoms, including reductions in pain, numbness and muscle weakness, with the use of a combination therapy involving MC and ALA (Maladkar et al., 2014). Moreover, this combination has been shown to improve nerve conduction velocity, an important measure of nerve function, suggesting that it not only alleviates symptoms but may also contribute to the regeneration of nerve tissues (Cai et al., 2012). A meta‐analysis further supports the superiority of the MC and ALA combination over MC alone, highlighting its effectiveness in treating diabetic peripheral neuropathy (Xu et al., 2013).

When comparing ALA with other treatments, differences in efficacy suggest that treatment choice should be tailored to the symptoms of individual patients (Bureković et al., 2008).

Neurosensorial abnormalities can be found in DED. These abnormalities are primarily due to alterations in corneal nerves, which play a crucial role in sensory processing and maintaining ocular surface homeostasis. The interplay between corneal nerve dysfunction and DED symptoms highlights the importance of understanding neurosensory pathways in the pathophysiology of DED.

Patients with DED exhibit significant alterations in corneal nerve structure, including reduced nerve density and increased tortuosity. These changes are more pronounced in aqueous‐deficient DED compared to evaporative DED, suggesting a direct link between nerve alterations and tear film deficiencies (Cox et al., 2021); (Guerrero‐Moreno et al., 2021). In the case of DED secondary to surgery, when confocal microscopy is performed, nerve amputations can be identified, with aberrant regeneration, nerve beading reduction and formation of micro neuromas. These findings are also related to cases of neuropathic pain after refractive surgery (Jing et al., 2022).

Corneal nerves in DED patients show altered responses to stimuli, contributing to symptoms such as pain and discomfort. These functional changes are associated with both peripheral and central sensitization mechanisms (Guerrero‐Moreno et al., 2021); (Kovács et al., 2016).

DED can be the origin or trigger of neuropathic pain. This is characterized by spontaneous pain, hyperalgesia and allodynia, which are not always correlated with the severity of ocular surface damage (Galor et al., 2022); (Ebrahimiadib et al., 2020).

The trigeminal nerve innervates the cornea and is crucial for processing sensory information. Dysfunction in this system can lead to chronic pain and photophobia, as seen in DED patients (Patel & Sarantopoulos, 2023); (Levitt et al., 2017). In addition, abnormal activity of corneal cold thermoreceptors has been implicated in the unpleasant sensations associated with DED. These receptors become hyperactive in response to chronic dryness, contributing to the neuropathic pain profile of the disease (Kovács et al., 2016).

DED is also associated with injury and inflammation. Chemical and pro‐inflammatory mediators, together with the intercellular communication of the immune response, activate the ion channels, increasing the sensitivity of the nociceptors, releasing an increase in peripheral stimuli response. Sustained inflammation conditions affect nervous activity and excitability. Neurogenic inflammation can produce nerve damage (peripheral sensitization) with aberrant activation and alteration of synaptic transmission (pain) (Baudouin et al., 2016); (Baudouin et al., 2018). Persistent activity in peripheral nerves, with sustained release of neurotransmitters, can lead to central sensitization and neuron–glial cell interaction, important for chronic pain.

The underlying mechanisms linking diabetic cornea and dry eye involve a complex interplay of factors including metabolic dysregulation, neurotrophic deficits, ocular surface inflammation and microbiota dysbiosis. Changes in tear composition and ocular surface anatomy collectively contribute to the onset and progression of DED in diabetic individuals (Zou et al., 2020); (Frida, 2017). In diabetic patients, the accumulation of advanced glycation end‐products (AGEs) and impaired neurotrophic innervations contribute significantly to diabetic keratopathy, characterized by delayed corneal epithelial wound healing and reduced corneal nerve density, which in turn affect tear secretion and ocular surface health (Zhou et al., 2022). The pathogenesis of diabetic DED is further complicated by the abnormal mitochondrial metabolism in the lacrimal gland, driven by the overactivation of the sympathetic nervous system, leading to reduced tear secretion and ocular discomfort (An & Zou, 2023). Recent studies have highlighted the role of ocular surface microbiota dysbiosis in diabetic patients, suggesting that a high‐glucose environment on the ocular surface can cause significant shifts in the microbiota composition. This dysbiosis promotes ocular surface inflammation and alters tear composition, disturbing the homeostasis of the ocular surface and exacerbating DED (An & Zou, 2023); (Toppo et al., 2022). Additionally, the structural and functional changes in the lacrimal gland, conjunctiva and meibomian glands due to diabetes contribute to the abnormal tear film and ocular surface disorders (Wan et al., 2022). MGD, a leading cause of DED, is exacerbated by diabetes through inflammatory pathways and microbiological changes, creating a vicious cycle of tear film instability and ocular surface damage (Baudouin et al., 2016). Inflammatory mediators and the activation of the Nod‐like receptor protein 3 inflammasome in diabetic patients further aggravate DED by promoting inflammation and ocular surface injury (Zhao & Zhang, 2022). Proteomic studies have identified differential expression of tear proteins in diabetic patients with DED, implicating inflammation, immune response and lipid metabolism in the pathogenesis (Ferdousi et al., 2018).

ALA has been studied for its potential benefits in treating DED in diabetic patients. Studies suggest that the administration of ALA in diabetic dry eye improves tear film parameters, reduces corneal defects and enhances antioxidant status (Ajith, 2020); (Roszkowska et al., 2022). ALA has shown potential in the prevention and treatment of diabetic retinopathy and keratopathy. ALA acts as a potent antioxidant and has been found to improve glycaemic control, insulin sensitivity and alleviate diabetic complications such as neuropathy and cardiovascular diseases (Bierbrauer et al., 2023). In terms of diabetic retinopathy, ALA has been shown to protect retinal cells, reduce inflammation and inhibit oxidative stress (Ajith, 2020); (Li et al., 2023). Some studies have shown that ALA supplementation may be useful in preventing vision loss and may help stabilize the development of diabetic retinopathy and prevent diabetic macular oedema (Nebbioso et al., 2013). Moreover, supplementation with ALA, along with genistein and vitamins, has shown promise in protecting retinal cells and reducing inflammation in pre‐retinopathic diabetic patients (Ajith, 2020).

In a prospective study performed in patients affected by type 2 diabetes with signs of distress and/or dry eyes, it was observed that treatment with ALA induces physiological recovery of the production of the tear film (Chisari et al., 2014). These findings suggest that ALA may be a promising treatment option for dry eye in diabetes and other ocular diseases associated with oxidative stress and inflammation (Li et al., 2023).

Additionally, ALA has been found to reduce the accumulation of AGEs and inhibit AGE receptor (RAGE) axis‐mediated oxidative stress, apoptosis and inflammation in diabetic keratopathy (Jwad et al., 2020). These findings suggest that ALA could be a promising treatment option for diabetic retinopathy and keratopathy (Buonfiglio et al., 2024).

Diabetes may also impact corneal nerves and lead to corneal neuropathy, which can lead to tear abnormalities due to inappropriate sensing and response to afferent signals (Zhang et al., 2023). Corneal confocal microscopy (IVCM) is a non‐invasive ophthalmic imaging technique that provides images of the cornea at the cellular level. Despite the uses in ocular surface pathologies, in the last decades, IVCM has been used to provide more knowledge in neuropathies diagnosis; IVCM is a very useful tool for identifying small nerve fibre damage in various peripheral neuropathies. IVCM has good reproducibility and is a useful diagnostic tool for screening some peripheral neuropathies, such as diabetic neuropathy (Cañadas et al., 2022).

ALA's effects on insulin sensitivity and secretion further underscore its utility in managing diabetic polyneuropathy, polycystic ovary syndrome and obesity (Capece et al., 2022). Clinical trials have shown that both parenteral and oral supplementation of ALA can improve neuropathic symptoms and deficits, with parenteral supplementation showing improvements over a 3‐week period and oral treatment having a more variable outcome (Bregovskiĭ et al., 2005). Clinical studies have further confirmed that ALA supplementation in patients with type 2 diabetes mellitus and diabetic neuropathy leads to a reduction in neurologic deficit and positive neurologic symptoms, alongside improvements in lipid spectrum and glycaemic control (Uryasyev et al., 2023). In patients with diabetic neuropathy, ALA treatment has led to significant improvements in sensory deficiency, particularly in those with a short history of diabetes mellitus and mild initial neurological disorders (Ametov et al., 2003).

### 3.3 ALA in ocular surface disorders

ALA has shown potential benefits in the treatment of DED. Oxidative stress is a significant factor in the development of DED triggered by ROS production, disrupting the healthy tear film, reducing the production of tears and leading to corneal damage. In addition, unopposed ROS can directly damage structures such as the tear lipid layer, as well as the myelin sheath of ocular surface nerves, leading to various processes that propagate dry eye (Seen & Tong, 2018).

Preclinical studies in animal models have demonstrated that ALA supplementation can mitigate oxidative stress‐induced damage to the ocular surface of DED, altering the metabolism of reactive nitrogen species and causing increased activity of lacrimal peroxidase and improved lacrimal production (Sarezky et al., 2016); (Andrade et al., 2014).

The protective role of ALA in DED involves several mechanisms. Oxidative stress triggers the activity of matrix metalloproteinase (MMP)‐2 and MMP‐9, leading to corneal epithelial degradation (Lee et al., 2010). An altered balance between MMP and their inhibitors in tears was one of the pathophysiological mechanisms associated with diseases of the ocular surface (Pflugfelder et al., 2017); (Ma et al., 2019). ALA can downregulate the expression of MMP‐2 and MMP‐9 in corneal epithelial cells and activate the antioxidant status of the ocular surface, preventing dry eye (Ajith, 2020); (Cavdar et al., 2014).

Tear proteins, such as lactoferrin and ascorbic acid, can be activated by ALA and contribute to the antioxidant defence of the ocular surface (Seen & Tong, 2018); (Ajith, 2020). Furthermore, ALA facilitates the release of nuclear factor erythroid‐2–related factor 2 (Nfr‐2) from its regulator Keaplike ECH‐associated protein 1 (Keap 1), enabling its migration into corneal epithelial cell nuclei (Andrade et al., 2014); (Kim et al., 2019). This process enhances the expression of antioxidant genes, elevating levels of superoxide dismutase (SOD), catalase (CAT) and glutathione peroxidase (GPx). These antioxidant enzymes, SOD, CAT and GPx, are present in the tissue of the ocular surface and tear film to maintain the integrity of the corneal epithelium and lacrimal unit (Behndig et al., 2001); (Alio et al., 1995) and are present at reduced levels in the conjunctival epithelium of patients with DED (Cejková et al., 2008).

Additionally, a potential role of nuclear factor of activated T cells 5 (NFAT5) and nuclear factor kappa‐light‐chain‐enhancer of activated B cells (NF‐κB) in the proinflammatory effect in the lacrimal gland and cornea is suggested. ALA has been found to have a protective effect on radiation‐induced lacrimal gland injuries, which can cause DED by reducing both NFAT5 and NF‐κB (Kim et al., 2019).

In a study on diabetic patients with dry eye symptoms, the combination of ALA and hydroxy‐propyl‐methylcellulose (HPMC) eye drops resulted in significant improvements in tear film break‐up time (TBUT), Ocular Surface Disease Index score, tear film morphology and corneal staining compared to HPMC alone (Roszkowska et al., 2022). Another study evaluated patients with DED after 90 days of topical use of ALA, showing that ALA eye drops elongate TBUT in the evaluated group of DED patients, improving tear stability (Stopyra, 2019). These findings suggest that ALA may be helpful in preventing or treating OSD in patients with DED.

**FIGURE 3:**
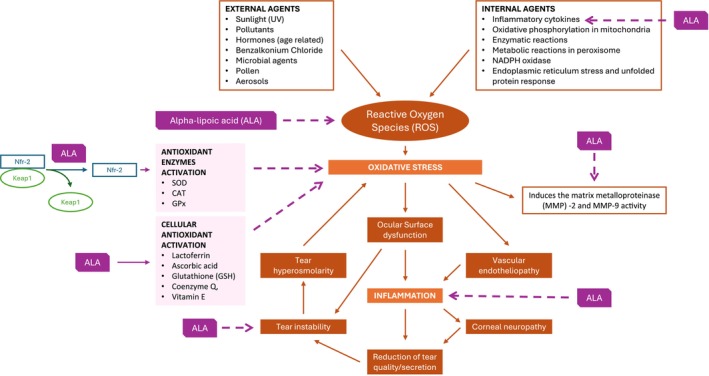
Therapeutic effects of ALA in DED through its potent antioxidant, anti‐inflammatory and neuroprotective properties.

ALA mechanism of action in DED is summarized in Figure 3.

ALA's antioxidative properties are particularly relevant in the context of MGD, as oxidative stress has been implicated in its pathogenesis and dysfunction. By scavenging free radicals and modulating redox status within the meibomian glands, ALA may mitigate oxidative damage, thereby preserving glandular function and reducing inflammation (Asiedu, 2022).

Chronic inflammation is a hallmark of MGD, contributing to structural changes in the meibomian glands and impairing their secretory function (Suzuki, 2018). ALA's anti‐inflammatory effects, attributed to its modulation of inflammatory signalling pathways and cytokine production, hold promise in attenuating glandular inflammation and restoring normal meibum secretion.

Beyond its antioxidative and anti‐inflammatory actions, ALA's cytoprotective properties (Diesel et al., 2007) may confer resilience to meibomian gland epithelial cells, safeguarding them from the harmful effects of oxidative stress and inflammatory mediators. ALA's ability to enhance cellular antioxidant defences and facilitate mitochondrial function may contribute to the preservation of glandular integrity in MGD.

Well‐designed clinical trials are warranted to ascertain the optimal dosing regimens and formulations of ALA, evaluate its long‐term efficacy and elucidate its precise mechanisms of action in managing MGD. Moreover, investigations into potential synergistic effects of ALA with conventional MGD therapies and its impact on clinical parameters and patient‐reported outcomes are essential for establishing its clinical utility.

Fuelled by its multifaceted pharmacological properties, ALA emerges as a promising adjunctive therapy for MGD, offering potential benefits through its antioxidative, anti‐inflammatory and cytoprotective effects. As research in this area progresses, ALA holds promise as a valuable therapeutic option in the armamentarium for managing MGD, addressing not only the underlying pathophysiological mechanisms but also enhancing the overall management of DED.

## 4 DISCUSSION

The main objective of this review is to summarize studies exploring the therapeutic use of ALA in treating or preventing specific ocular diseases and its role in neurosensorial abnormalities, but ALA could also have significant therapeutic potential due to its potent antioxidant and anti‐inflammatory properties. It is involved in various metabolic processes and has been studied for its role in managing multiple health conditions, particularly those associated with oxidative stress and inflammation. In this way, the applications of ALA in conditions such as diabetic neuropathy (Fasipe et al., 2023); (Nguyen et al., 2024), cardiovascular and metabolic disorders (Wollin & Jones, 2003); (Huerta et al., 2019), cancer (Abdullah et al., 2024); (Choi et al., 2021), neurological disorders (de Sousa et al., 2019); (Seifar et al., 2019), Covid‐19 (Sayıner et al., 2021), obesity (Namazi et al., 2018), rheumatoid arthritis and multiple sclerosis (Huerta et al., 2019), among other pathologies, are being studied; therefore, the present review does not cover some significant applications of ALA.

Due to its unique properties and mechanisms of action, ALA could be considered superior to other antioxidants in treating OSD. ALA's ability to regenerate endogenous antioxidants, its metal chelating activity and its capacity to directly scavenge ROS makes it particularly effective in addressing oxidative stress‐related ocular conditions. Unlike some endogenous antioxidants that are present in low concentrations, ALA can be administered exogenously, providing a more substantial antioxidant effect. This is particularly beneficial in conditions where endogenous antioxidant levels are insufficient (Packer, 1994); (Packer et al., 2001). The AREDS study highlighted the benefits of a combination of antioxidants in slowing AMD progression. ALA's unique properties, such as its ability to regenerate other antioxidants, suggest it could enhance the efficacy of such combinations (Kowluru & Zhong, 2011). ALA's dual role as an antioxidant and a metal chelator further distinguishes it from other antioxidants, providing additional protective mechanisms against oxidative stress (Malińska & Winiarska, 2005).

The review also captured a few data on ALA dosage, forms of administration (oral vs. topical) from studies focusing on ocular health. In studies related to diabetic neuropathy, ALA has been administered both intravenously and orally. Intravenous administration of 600 mg daily over 3 weeks has shown significant improvements in neuropathic pain, suggesting a similar dosing strategy could be effective for ocular conditions associated with neuropathy (Mijnhout et al., 2010). Oral administration of 800 mg daily for 4 months has been used in studies for cardiac autonomic neuropathy, indicating that higher doses over extended periods might be necessary for chronic conditions (Ziegler & Gries, 1997). There is a notable gap in research specifically addressing the optimal topical doses of ALA for ocular diseases. Further studies are needed to establish effective concentrations and formulations for topical application. Combining both routes could potentially enhance therapeutic outcomes, utilizing the strengths of each method.

While ALA's therapeutic potential is significant, its efficacy is often limited by its pharmacokinetic profile, including a short half‐life and low bioavailability. To overcome ALA's limitations, nanotechnology has been employed. Various nanoparticulate systems, including lipid‐based nanoparticles and polymeric nanoparticles, have been developed to enhance ALA's stability, solubility and targeted delivery (Bellini et al., 2024); (Mosallaei et al., 2024). Nanoformulations of ALA have shown promise in specific applications, such as skin care, ophthalmic treatments and cerebral ischemia, by providing sustained release and improved absorption (Mosallaei et al., 2024). The engineering of poly(α‐lipoic acid) nanoparticles offers a promising drug delivery system, potentially enhancing the therapeutic effectiveness of ALA (Bellini et al., 2024).

Other innovative formulations, such as liquid and amphiphilic matrices, have been developed, leading to improved absorption and therapeutic outcomes (Salehi et al., 2019).

Additionally, ALA's effectiveness may vary with age and its role as adjuvant therapy in various conditions continues to be explored (Khaibulina et al., 2024).

Despite these challenges, the ongoing research and development of advanced delivery systems hold promise for expanding ALA's therapeutic applications across a broader spectrum of pathologies.

## 5 CONCLUSIONS

ALA, with its antioxidant and anti‐inflammatory effects, presents a promising avenue for managing DED.

ALA mitigates oxidative stress‐induced damage and improves tear film stability and ocular surface health.

It also has potential benefits in ocular complications associated with diabetes and neurological disorders.

Despite the clinical and preclinical studies, more targeted long‐term clinical trials using different oral doses or eye drops of ALA are warranted to explore their therapeutic potential in ocular diseases.

Further research is needed to determine optimal dosing regimens, safety profiles and efficacy in different patient populations.

## AUTHOR CONTRIBUTIONS

Antonio J. Mateo Orobia has participated in conceptualization, methodology and validation. All the authors have participated in the investigation, resources, data curation, writing—original draft preparation, writing—review and editing. All the authors have read and agreed to the published version of the manuscript.

## FUNDING INFORMATION

This review has not received funding.

## CONFLICT OF INTEREST STATEMENT

The authors declare no conflict of interest.

## INSTITUTIONAL REVIEW BOARD STATEMENT

The study was conducted following the Declaration of Helsinki.
